# In-Plane Vibration Analysis of Rectangular Plates with Elastically Restrained Boundaries Using Differential Quadrature Method of Variational Weak Form

**DOI:** 10.3390/ma18143250

**Published:** 2025-07-10

**Authors:** Xianke Wang, Weipeng Zhou, Shichao Yi, Sen Li

**Affiliations:** 1 School of Science, Jiangsu University of Science and Technology, Zhenjiang 212003, China; 231110502102@stu.just.edu.cn (X.W.); shichaoyi@just.edu.cn (S.Y.); 2 School of Naval Architecture and Ocean Engineering, Jiangsu University of Science and Technology, Zhenjiang 212003, China; 3 Zhenjiang Jizhi Ship Technology Co., Ltd., Zhenjiang 212003, China; 4 Yangzijiang Shipbuilding Group, Taizhou 212299, China; 5 School of Science, Changzhou Institute of Technology, Changzhou 213032, China; lisen@czu.cn

**Keywords:** variational principles, DQM, weak form equations, elastically restrained boundaries, in-plane vibrational behavior

## Abstract

An efficient numerical approach utilizing a variational weak form, grounded in 2D elastic theory and variational principles, is proposed for analyzing the in-plane vibrational behavior of rectangular plates resting on elastically restrained boundaries. The differential and integral operators can be discretized into matrix representations employing the differential quadrature method (DQM) and Taylor series expansion techniques. The discretization of dynamics equations stems directly from a weak formulation that circumvents the need for any transformation or discretization of higher-order derivatives encountered in the corresponding strong equations. Utilizing the matrix elementary transformation technique, the displacements of boundary and internal nodes are segregated, subsequently leading to the derivation of the generalized eigenvalue problem pertaining to the free vibration analysis of the Functionally Graded Material (FGM) rectangular plate. Furthermore, the study examines the impact of the gradient parameter, aspect ratio, and elastic constraints on the dimensionless frequency characteristics of the FGM rectangular plate. Ultimately, the modal properties of an in-plane FGM rectangular plate are investigated.

## 1. Introduction

As a fundamental component of various structures, the plate structure finds extensive application in aerospace, shipbuilding, construction, and numerous other industries. The primary focus of research pertaining to the dynamic characteristics of plates revolves around bending vibration or low-frequency oscillations, with a keen emphasis on investigating the vibrational behavior of plates of diverse shapes subject to various boundary conditions. Conversely, research into the in-plane vibration of plates remains relatively scarce. The in-plane vibration of the plate not only transmits high-frequency vibrations and energy but also exerts an influence on the low-frequency vibrations [1,2,3]. The application of high-frequency vibration in plate structures within engineering contexts, including operational scenarios, underscores the importance of studying in-plane vibration characteristics of these structures.

Kobayashi et al. [4] employed the Ritz method to scrutinize the frequency and modal attributes of the in-plane vibration exhibited by a rectangular plate, taking into account varying boundary conditions pertaining to different points of support. Shu et al. [5] developed a generalized approach (CBCGE) for implementing general boundary conditions in the GDQ free vibration analysis of rectangular plates by directly coupling boundary conditions with governing equations, and applied it to plates with various combinations of free edges and corners. Fuchs et al. [6] presented a unified solution for rectangular plates with elastic corner and boundary supports by evaluating constants via operational formulas, and applied it to beams—supported simply by plates under uniform load. Gutierrez et al. [7] assumed that the in-plane displacement of the rectangular plate satisfies the superposition of the boundary conditions of the triangular series, and used the Ritz method to solve the in-plane vibration frequency of a rectangular plate that is normally elastically constrained and tangentially free, and discussed the effect of elastic constraint stiffness on frequency. Bardell et al. [8] used the Ritz method to solve the frequency characteristics and modal characteristics of a rectangular plate under three boundary conditions: simply supported, fixed, and free. Farag and Pan [9] derived in-plane free and forced vibration differential equations for rectangular plates based on thin-plate theory and hysteresis damping model, and solved the dynamic responses under in-plane harmonic excitation, considering the longitudinal and shear waves of in-plane vibrations. On this basis, Farag and Pan [10] solved the in-plane free vibration characteristics and modal characteristics of a rectangular plate with fixed constraints on opposite sides, gave the modal response function of the rectangular plate under in-plane excitation, and discussed three modal characteristics of forced vibration in a rectangular plate under various boundary conditions. Wang et al. [11] solved the in-plane vibration frequency and modal characteristics of rectangular plates based on the Kantorovich–Krylov variational method, and discussed the in-plane vibrations of C-C-C-C, C-C-C-F, and C-F-C-F rectangular plates. Du et al. [12] used the improved Fourier series method to analyze the free vibration frequency in the rectangular plate under the condition of four-side elastic constraint, and by controlling the stiffness coefficient of the elastic constraint, they discussed the frequency and the aspect ratio of the rectangular plate under various boundary conditions. Variation law and modal characteristics. On this basis, the in-plane free vibration of a rectangular plate under elastic support at a point on the boundary is further studied [13]. Gorman [14] analyzed the in-plane vibration of a rectangular plate by using the superposition principle, and solved the in-plane vibration frequency of the rectangular plate in the case of symmetry and anti-symmetry. On this basis, the in-plane vibration frequencies and modal characteristics of the C-C-C-C and S-S-S-S rectangular plates are further solved [15], and it is pointed out that there are pure longitudinal vibration and pure shear modes under the simply supported boundary, and the normal elastic constraints are further solved. Furthermore, the in-plane vibration problem of a tangentially free rectangular plate [16] and the in-plane vibration frequency and model characteristics of a rectangular plate with two pairs of sides simply supported are also solved [17]. Xing et al. [18] derived the in-plane free vibration differential equation of a rectangular plate based on the Rayleigh variational principle, solved the differential equation by directly separating the variables, and discussed the frequency characteristics and modal characteristics of the two pairs of simply supported rectangular plates. The problem of frequency loss and duplication is presented in [18]. Dozio [19] solved the in-plane vibration of an isotropic rectangular plate under arbitrary non-uniform elastic boundary conditions based on the Ritz method and the displacement assumption of trigonometric functions, and discussed the effect of non-uniform elastic stiffness on frequency. Chen et al. [20] solved the bending and in-plane vibration of a notched rectangular plate using truncated Chebyshev polynomials. Shi et al. [21] used an improved Fourier series method to solve the in-plane vibration of an anisotropic rectangular plate with non-uniform elastic support on the boundary and straight internal support.

In recent years, new trends have emerged in the in-plane vibration of plate structures: A series of theoretical methods of unified solution were successively applied to various vibration problems. Chen et al. first proposed a unified solution under a semi-analytical solution framework for the in-plane free vibration analysis of elastic functionally graded porous plates (FGPPs) with porosity distributions in the thickness and in-plane directions [22]. In [23], a unified solution was proposed for the in-plane vibration analysis of composite laminated sector plates and annular plates with elastic constraints. By means of the improved Fourier series method (IFSM), a unified formula was used to analyze the in-plane free vibration of arbitrarily shaped straight-sided quadrilateral and triangular plates with arbitrary boundary conditions in [24]. The isogeometric model was applied to the in-plane free vibration problem. In the paper [25], non-uniform rational Lagrangian functions were used in the isogeometric analysis of in-plane vibration and flexural vibration of thin plates. Xue et al. investigated the free vibration behaviors of functionally graded (FG) plates, considering in-plane material inhomogeneity using the isogeometric approach [26]. The isogeometric three-dimensional vibration scheme was used to study variable thickness parallelogram plates with in-plane functionally graded porous materials in [27]. The research on in-plane vibration problems witnessed significant progress. Subsequently, people delved deeper into the in-plane vibration problems of variously shaped plates, such as sector-shaped, annular, porous, and arbitrarily shaped plates. By referring to the Helmholtz decomposition theorem, the model of the elliptical thin plate was simplified. Then, using the method of separation of variables in elliptical coordinates, the decoupled governing equations were solved based on the product of (even and odd) angular and radial Mathieu functions, thus resolving the free tensile vibration problem of the elliptical thin plate [28]. Baferani et al. presented the exact analytical solutions for the vibration and buckling of symmetrically laminated thick rectangular plates subjected to different types of in-plane loads on an elastic foundation in [29]. Wang et al., in their research [30], proposed a unified approach for the vibration analysis of functionally graded circular plates, annular plates, and annular circular sector plates with general boundary conditions, based on the first-order shear deformation theory and the Ritz method. In the paper [23], a semi-analytical method for the in-plane vibration analysis of a composite laminated annular and sector plate with elastic constraints was presented. Kamranfard et al. constructed an analytical solution for the vibration and buckling of annular sectorial porous plates under in-plane uniform compressive loading in [31]. Zhong et al. predicted the in-plane vibration characteristics of the porous annular plate with porosity distributions in the thickness and radial directions in the article [32]. Arbitrarily shaped plates with curvilinear geometry were first applied to the free in-plane vibration using the spectral Chebyshev method under different boundary conditions in [33]. Recently, the research of most scholars shifted to the study of problems of plates with complex structures. Correspondingly, the problems of laminated plates were solved. Tornabene et al. [34] developed a general formulation for the dynamic analysis of anisotropic laminated doubly curved shells with arbitrary shapes, variable thickness, and general boundary conditions, employing the GDQ method within a higher-order ESL framework. Baferani et al, Hashemi et al., and Lal et al. successively used the first-order shear deformation theory, Reddy’s third-order shear deformation theory, and classical plate theory to study the in-plane vibration of plate structures in articles [29,35,36]. At that time, the theory based on the 3D model was a hot topic issue. Pagani et al. presented a novel numerical approach for studying the vibration behaviors of variable angle tow (VAT) composite structures in their quasi-static nonlinear equilibrium states in the work [37]. Pu et al. [38,39] used the differential quadrature method to solve the in-plane free vibration of the isotropic rectangular plate and the functionally graded rectangular plate.

To sum up, there are many articles on the in-plane free vibration of uniform rectangular plates, but there are few studies on the in-plane vibration of in-plane functionally graded plates. Therefore, on the basis of its predecessors, this paper uses the variational weak DQM method to study the in-plane free vibration of the in-plane bidirectional functionally graded rectangular plate, and reveals the in-plane longitudinal vibration and in-plane shear in the in-plane vibration of the rectangular plate. According to the coupling characteristics of vibration, it can be concluded that there are universal laws that can be followed, so as to have a guiding role in practical engineering applications.

## 2. Basic Equation

### 2.1. Establishment of Variational Equations for In-Plane Free Vibration

**Figure 1:**
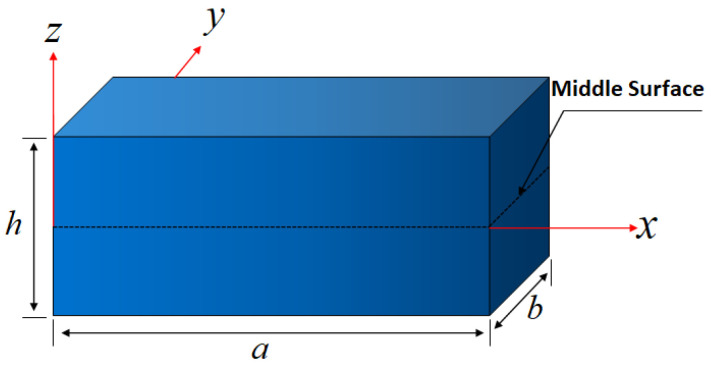
Schematic diagram of in-plane graded plate.

Considering the structural model of a rectangular plate composed of a metal–ceramic in-plane functionally graded material (FGM), as depicted in Figure 1, the dimensions of this FGM plate are defined by its length *a*, width *b*, and thickness *h*. The four boundaries of this plate are specified (x=0→y=0→x=a→y=b). The springs are evenly distributed in both the normal and tangential directions, with their respective normal and tangential stiffnesses denoted as $k_{n}$ and $k_{\tau }$.

**Figure 2:**
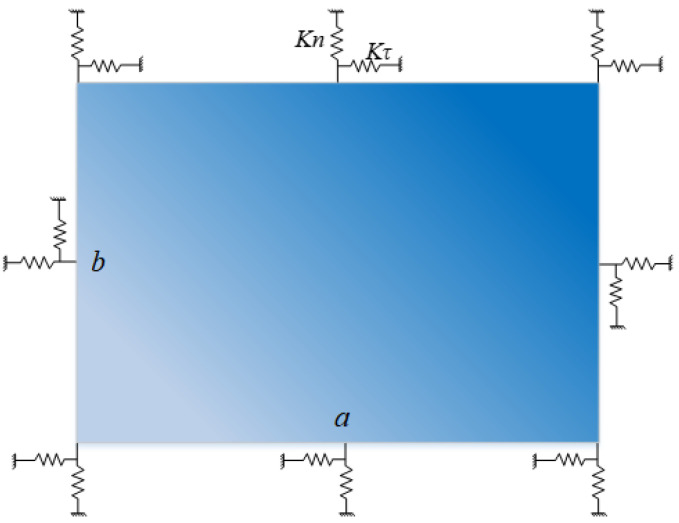
A rectangular plate resting upon elastic restraint boundaries.

The FGM rectangular plate exhibits a continual gradient in its material properties along the in-plane direction in Figure 2, with its mass density denoted as ρ, Poisson’s ratio as μ, and elastic modulus as *E*, all adhering to a power function variation.

(1) $$P\left(x,y\right)=P_{m}+\left(P_{c}-P_{m}\right)V_{c},$$

where $V_{c}$ represents the volume fraction of ceramics. The volume fraction $V_{c}$ varies unidirectionally along the axis y

(2) $$V_{c}={\left(\frac{y}{b}\right)}^{p},$$

or has a *x*, *y* bidirectional change along the axis

(3) $$V_{c}={\left(\frac{xy}{ab}\right)}^{p}.$$

Let *x* the displacement components of the direction and the direction be *y*, respectively; *u*, *v* and the geometric equation is

(4) $$\varepsilon ={\left[\varepsilon_{x},\varepsilon_{y},\gamma_{xy}\right]}^{T}=\mathrm{LU},$$

in

(5) $$L=\left[\begin{array}{cc} \frac{\partial }{\partial x} & 0\\ 0 & \frac{\partial }{\partial y}\\ \frac{\partial }{\partial y} & \frac{\partial }{\partial x} \end{array}\right],U=\left[\begin{array}{c} u\\ v \end{array}\right].$$

$\varepsilon_{x}$ and $\varepsilon_{y}$ represent the normal strain in the directions *x* and *y*, respectively, while $\gamma_{xy}$ denotes the shear strain.

The physical equation is

(6) $$\sigma ={\left[\sigma_{x},\sigma_{y},\sigma_{xy}\right]}^{T}=C\varepsilon$$

where $\sigma_{x}$ and $\sigma_{y}$ represent the normal stresses in the *x*-direction and *y*-direction, respectively, $\sigma_{xy}$ represents the shear stress, and **C** is the stiffness matrix.

(7) $$C=\left[\begin{array}{ccc} c_{11} & c_{13} & 0\\ c_{13} & c_{33} & 0\\ 0 & 0 & c_{55} \end{array}\right].$$

For isotropic material plates

$$c_{11}=c_{33}=\frac{E(x,y)}{1-\mu^{2}\left(x,y\right)},c_{13}=\frac{\mu (x,y)E(x,y)}{1-\mu^{2}\left(x,y\right)},c_{55}=\frac{E(x,y)}{2\left(1+\mu (x,y)\right)},$$

E(x,y),μ(x,y) are Young’s modulus and Poisson’s ratio, respectively. Kinetic energy and strain energy of the rectangular plate are Young’s modulus and Poisson’s ratio, respectively.

Kinetic energy and strain energy of the rectangular plate are

(8) $$\begin{array}{c} \mathrm{II}=\frac{1}{2}\int_{\Omega }\varepsilon^{T}\sigma d\Omega ,T=\frac{1}{2}\int_{\Omega }{\dot{U}}^{T}\rho \dot{U}d\Omega \end{array}.$$

### 2.2. Matrixing of Differential and Integral Operators

Let us consider a one-dimensional problem as an illustrative example, specifically by assuming a column vector

(9) $$U={\left[\begin{array}{cc} u\left(x_{1}\right) & u\left(x_{2}\right)\cdots u\left(x_{n}\right) \end{array}\right]}^{T},$$

where $u(x_{i})$ represents the function value of the first *i* node, and *n* denotes the total number of nodes.

Utilizing the differential quadrature method for the discretization of the r-order derivative of Formula (9) at the node, we can represent $U^{\left(r\right)}$ in a discrete form.

(10) $$U^{\left(r\right)}=A^{\left(r\right)}U,$$

where $A^{\left(r\right)}$ represents the r-order differential quadrature matrix

(11) $$A_{ij}^{\left(r\right)}=\left\{\begin{array}{cc} I_{ij}, & r=0;\\ \frac{\prod_{k=1,k\neq i}^{n}\left(x_{i}-x_{k}\right)}{\left(x_{i}-x_{j}\right)\prod_{k=1,k\neq j}^{n}\left(x_{j}-x_{k}\right)}, & i\neq j,r=1;\\ r\left(A_{ij}^{\left(1\right)}A_{ii}^{\left(r-1\right)}-\frac{A_{ij}^{\left(r-1\right)}}{x_{i}-x_{j}}\right), & i\neq j,r=2,\cdots ,n-1;\\ -\sum_{j=1,j\neq i}^{n}A_{ij}^{\left(r\right)}, & i=j,r=1,2,\cdots n-1. \end{array}\right.$$

Using Taylor series, we expand the function u(x) at points $x_{i}$

(12) $$u\left(x\right)={\left.\sum_{r=0}^{\infty }\frac{{\left(x-x_{i}\right)}^{r}}{r!}\frac{d^{r}u}{dx^{r}}\right|}_{x=x_{i}}.$$

The integral of Equation (12) evaluated over the interval $\left[\frac{x_{i-1}+x_{i}}{2},\frac{x_{i}+x_{i+1}}{2}\right]$ can be formulated as follows:

(13) $$\int_{\frac{x_{i-1}+x_{i}}{2}}^{\frac{x_{i}+x_{i+1}}{2}}u\left(x\right)dx={\left.\sum_{r=0}^{\infty }\frac{{\left(x_{i+1}-x_{i}\right)}^{r+1}-{\left(x_{i-1}-x_{i}\right)}^{r+1}}{2^{r+1}\left(r+1\right)!}\frac{d^{r}u}{dx^{r}}\right|}_{x=x_{i}}.$$

Therefore, to compute the integral encompassing the entire area from $\left[\begin{array}{c} x_{1},x_{n} \end{array}\right]$,

(14) $$\begin{array}{cc} \int_{x_{1}}^{x_{n}}u\left(x\right)dx & =\sum_{r=0}^{\infty }\left(\frac{{\left(x_{2}-x_{1}\right)}^{r+1}}{2^{r}\left(r+1\right)!}\frac{d^{r}u}{dx^{r}}{|}_{x_{1}}\right.\\ \; & +\sum_{i=2}^{n-1}\frac{{\left(x_{i+1}-x_{i}\right)}^{r+1}-{\left(x_{i-1}-x_{i}\right)}^{r+1}}{2^{r+1}\left(r+1\right)!}\frac{d^{r}u}{dx^{r}}{|}_{x_{i}}\\ \; & \left.-\frac{{\left(x_{n-1}-x_{n}\right)}^{r+1}}{2^{r}\left(r+1\right)!}\frac{d^{r}u}{dx^{r}}{|}_{x_{n}}\right), \end{array}$$

will be Equation (14) written in matrix form

(15) $$\int_{x_{1}}^{x_{n}}u\left(x\right)dx=\sum_{r=0}^{\infty }{\tilde{X}}^{\left(r\right)}U^{\left(r\right)},$$

in

(16) $$\begin{array}{cc} {\tilde{X}}^{\left(r\right)} & =\left[\frac{{\left(x_{2}-x_{1}\right)}^{r+1}}{2^{r+1}\left(r+1\right)!},\cdots ,\frac{{\left(x_{i+1}-x_{i}\right)}^{r+1}-{\left(x_{i-1}-x_{i}\right)}^{r+1}}{2^{r+1}\left(r+1\right)!},\right.\\ \; & \;\cdots ,\left.-\frac{{\left(x_{n-1}-x_{n}\right)}^{r+1}}{2^{r}\left(r+1\right)!}\right],\;i=2,3,\cdots ,n-1. \end{array}$$

If we undertake an n−1 order expansion of Equation (15) and subsequently substitute Equation (10) into it, we obtain

(17) $$\int_{x_{1}}^{x_{n}}u\left(x\right)dx=\left(\sum_{r=0}^{n-1}{\tilde{X}}^{\left(r\right)}A^{\left(r\right)}\right)U=\tilde{S}U,$$

For two-dimensional problems, the partial derivative of the function can be articulated as

(18) $$\frac{\partial^{r_{1}+r_{2}}u}{\partial x^{r_{1}}\partial y^{r_{2}}}=\left(A_{x}^{\left(r_{1}\right)}⊗A_{y}^{\left(r_{2}\right)}\right)U,$$

in

(19) $$\begin{array}{c} U={\left[u_{11},\cdots ,u_{1n},u_{21}\cdots u_{mn}\right]}^{T} \end{array},$$

m,n are the number of x,y direction nodes, respectively, the subscript of the x,y matrix represents *A* the differential quadrature coefficient matrix in the direction, and “⊗” is the Kronecker tensor product.

(20) $$\int_{x_{1}}^{x_{m}}\int_{y_{1}}^{y_{n}}u\left(x,y\right)dxdy=\left({\tilde{S}}_{x}⊗{\tilde{S}}_{y}\right)U.$$

## 3. Discretization and Eigenvalue Problem

### 3.1. Discretization of Weak Form Equations

For a two-dimensional problem, assume a node matrix

(21) $$\bar{U}={\left[u_{H},\cdots ,u_{\mathrm{nar}},v_{H},\cdots v_{\mathrm{ner}}\right]}^{T}$$

Employing the DQM in conjunction with the integral operator matrix algorithm, the strain energy can be discretized in the subsequent refined form:

(22) $$\begin{array}{cc} \mathrm{II} & =\frac{1}{2}\int_{\Omega }\varepsilon^{T}\sigma d\Omega =\frac{1}{2}\int_{\Omega }U^{T}L^{T}C_{1}LUd\Omega \\ \; & =\frac{1}{2}\bar{S}\;\bar{L}\;\bar{U}\circ {\bar{C}}_{1}\;\bar{L}\;\bar{U}\\ \; & =\frac{1}{2}{\bar{U}}^{T}{\bar{L}}^{T}\left(I_{3}⊗S\right){\bar{C}}_{1}\bar{L}\;\bar{U} \end{array}$$

in

(23) $$L_{1}=A_{x}^{\left(1\right)}⊗I_{n},L_{2}=I_{m}⊗A_{y}^{\left(1\right)},\bar{L}=\left[\begin{array}{cc} L_{1} & 0\\ 0 & L_{2}\\ L_{2} & L_{1} \end{array}\right]$$

(24) $$\begin{array}{cc} {\bar{C}}_{1} & =\left[\begin{array}{ccc} {\bar{C}}_{11} & {\bar{C}}_{13} & \\ {\bar{C}}_{13} & {\bar{C}}_{33} & \\ & \; & {\bar{C}}_{55} \end{array}\right],{\bar{C}}_{11}={\bar{C}}_{33}=\mathrm{diag}\left(\mathrm{vec}\left({\bar{C}}_{1}^{11}\right)\right)\\ {\bar{C}}_{13} & =\mathrm{diag}\left(\mathrm{vec}\left({\bar{C}}_{1}^{13}\right)\right),{\bar{C}}_{55}=\mathrm{diag}\left(\mathrm{vec}\left({\bar{C}}_{1}^{55}\right)\right)\\ {\bar{C}}_{1}^{11} & =\left[\begin{array}{ccc} c_{11}\left(x_{1},y_{1}\right) & \cdots & c_{11}\left(x_{m},y_{1}\right)\\ \vdots & \ddots & \vdots \\ c_{11}\left(x_{1},y_{n}\right) & \cdots & c_{11}\left(x_{m},y_{n}\right) \end{array}\right]\\ {\bar{C}}_{1}^{13} & =\left[\begin{array}{ccc} c_{13}\left(x_{1},y_{1}\right) & \cdots & c_{13}\left(x_{m},y_{1}\right)\\ \vdots & \ddots & \vdots \\ c_{13}\left(x_{1},y_{n}\right) & \cdots & c_{13}\left(x_{m},y_{n}\right) \end{array}\right]\\ {\bar{C}}_{1}^{55} & =\left[\begin{array}{ccc} c_{55}\left(x_{1},y_{1}\right) & \cdots & c_{55}\left(x_{m},y_{1}\right)\\ \vdots & \ddots & \vdots \\ c_{55}\left(x_{1},y_{n}\right) & \cdots & c_{55}\left(x_{m},y_{n}\right) \end{array}\right] \end{array}$$

(25) $$\tilde{S}={\tilde{S}}_{x}⊗{\tilde{S}}_{y},\bar{S}=\left[\begin{array}{ccc} 1 & 1 & 1 \end{array}\right]⊗\tilde{S},S=\mathrm{diag}\left(\tilde{S}\right),\mathrm{diag}\left(\bar{S}\right)=I_{3}⊗S$$

The operator “∘” is defined as follows: When **a** represents a column vector, **b** and **c** are row vectors, and the operation proceeds as $a\left(b\circ c\right)=b^{T}diag\left(a\right)c$. Meanwhile, the operator “vec” signifies the process of vectorizing a matrix.

Analogously, kinetic energy can be discretized as

(26) $$\begin{array}{c} T=\frac{1}{2}\int_{\Omega }{\dot{U}}^{T}\rho \dot{U}d\Omega =\frac{1}{2}\int_{\Omega }{\dot{\bar{U}}}^{T}\rho \dot{\bar{U}}d\Omega \\ =\frac{1}{2}\bar{S}\left(\dot{\bar{U}}\circ \bar{\rho }\dot{\bar{U}}\right)=\frac{1}{2}\dot{\bar{U}}\left(I_{2}⊗S\right)\bar{\rho }\dot{\bar{U}} \end{array}$$

in

(27) $$\begin{array}{c} \rho =\left[\begin{array}{cc} \rho & \\ & \rho \end{array}\right],\rho_{1}=\left[\begin{array}{ccc} \rho \left(x_{1},y_{1}\right) & \cdots & \rho \left(x_{m},y_{1}\right)\\ \vdots & \ddots & \vdots \\ \rho \left(x_{1},y_{n}\right) & \cdots & \rho \left(x_{m},y_{n}\right) \end{array}\right]\\ {\bar{\rho }}_{1}=\mathrm{diag}\left(\mathrm{vec}\left(\rho_{1}\right)\right),\bar{\rho }=\left[\begin{array}{cc} {\bar{\rho }}_{1} & \\ & {\bar{\rho }}_{1} \end{array}\right] \end{array}$$

The vibrational equation can be derived by employing the variational principle in conjunction with the partial integration formula

(28) $$M\ddot{\bar{U}}+K\bar{U}=0$$

in

(29) $$\begin{array}{c} M=\left(I_{2}⊗S\right)\bar{\rho },K={\bar{L}}^{T}\left(I_{3}⊗S\right){\bar{C}}_{1}\bar{L} \end{array}$$

The matrices **M** and **K** can be represented in the following block form

(30) $$M=\left[\begin{array}{cc} M_{11} & M_{12}\\ M_{21} & M_{22} \end{array}\right],K=\left[\begin{array}{cc} M_{11} & M_{12}\\ M_{21} & M_{22} \end{array}\right]$$

The subsequent process primarily leverages matrix transformation techniques to isolate the displacement components pertinent to internal nodes and boundary points. Specifically, considering the case where m=n, we employ all internal nodes to construct the transformation matrix $Z_{i}\in R^{{\left(n-2\right)}^{2}\times n^{2}}$. In doing so,

(31) $$Z_{i}={\left[\begin{array}{ccccccc} e_{n+2} & \cdots & e_{2n-1} & e_{2n+1} & \cdots & e_{3n-1} & \cdots \end{array}\right]}^{T}$$

where $e_{i}\in R^{n^{2}\times 1}$ denotes the unit vector in which the *i* component is 1, with all remaining components being 0.

(32) $$Z_{b}={\left[\begin{array}{cccc} e_{1}\cdots e_{n} & e_{n+1} & e_{2n} & e_{2n+1}\cdots e_{n(n-1)+1}\cdots e_{n^{2}} \end{array}\right]}^{T}$$

Using matrix transformation, the dynamic Equation (28) can be transformed into

(33) $$L_{b}U_{b}+L_{i}U_{i}=\omega^{2}\left(M_{b}U_{b}+M_{i}U_{i}\right)$$

in

(34) $$\begin{array}{c} L_{b}=\left[\begin{array}{cc} Z_{i}K_{11}Z_{b}^{T} & Z_{i}K_{12}Z_{b}^{T}\\ Z_{i}K_{21}Z_{b}^{T} & Z_{i}K_{22}Z_{b}^{T} \end{array}\right]\\ L_{i}=\left[\begin{array}{cc} Z_{i}K_{11}Z_{i}^{T} & Z_{i}K_{12}Z_{i}^{T}\\ Z_{i}K_{21}Z_{i}^{T} & Z_{i}K_{22}Z_{i}^{T} \end{array}\right]\\ M_{b}=\left[\begin{array}{cc} Z_{i}M_{11}Z_{b}^{T} & Z_{i}M_{12}Z_{b}^{T}\\ Z_{i}M_{21}Z_{b}^{T} & Z_{i}M_{22}Z_{b}^{T} \end{array}\right]\\ M_{i}=\left[\begin{array}{cc} Z_{i}M_{11}Z_{i}^{T} & Z_{i}M_{12}Z_{i}^{T}\\ Z_{i}M_{21}Z_{i}^{T} & Z_{i}M_{22}Z_{i}^{T} \end{array}\right] \end{array}$$

(35) $$U_{i}=\left[\begin{array}{c} u_{i}\\ v_{i} \end{array}\right],U_{b}=\left[\begin{array}{c} u_{b}\\ v_{b} \end{array}\right]$$

### 3.2. Discrete Boundary Conditions

For a rectangular plate with elastic constraints on four sides (x=0,x=a,y=0,y=b), the stress expressions of boundary conditions are

$$\sigma_{x}=c_{11}\frac{\partial u}{\partial x}+c_{13}\frac{\partial v}{\partial y},\sigma_{y}=c_{13}\frac{\partial u}{\partial x}+c_{33}\frac{\partial v}{\partial y},\tau_{xy}=c_{55}\left(\frac{\partial u}{\partial y}+\frac{\partial v}{\partial x}\right).$$

The specific corresponding results are as follows: $\left(k_{rx0}u,-,k_{zx0}v\right)$ for x=0, $\left(-k_{rx1}u,-,-k_{rx1}v\right)$ for x=a, $\left(-,k_{ry0}v,k_{xy0}u\right)$ for y=0, and $\left(-,-k_{ry1}v,-k_{rx1}u\right)$ for y=b. Especially when $k_{n}$ and $k_{\tau }$ take some special values, they can degenerate into several common boundary conditions; the x=0 boundary can be taken as an example, with $k_{nx0}=\left(\infty ,0,0,\infty \right)$, and $k_{\tau x0}=\left(\infty ,0,\infty ,0\right)$ for four boundary conditions being as follows: fixed edge(C), free edge(F), special boundary 1(S1), special boundary 2(S2).

By utilizing the differential quadrature method, the displacement and its derivative at the boundary point x=0 can be formulated

(36) $$\begin{array}{c} u=\left(e_{1}^{T}⊗I_{n}\right)u,u_{x}=\left(e_{1}^{T}A_{x}^{\left(1\right)}⊗I_{n}\right)u,u_{y}=\left(e_{1}^{T}⊗A_{y}^{\left(1\right)}\right)u,\\ v=\left(e_{1}^{T}⊗I_{n}\right)v,v_{x}=\left(e_{1}^{T}A_{x}^{\left(1\right)}⊗I_{n}\right)v,v_{y}=\left(e_{1}^{T}⊗A_{y}^{\left(1\right)}\right)v \end{array}$$

Analogously, at the boundary where y=0, the boundary conditions can be formulated as

(37) $$\begin{array}{c} u=\left(I_{n}⊗e_{1}^{T}\right)u,u_{x}=\left(A_{x}^{\left(1\right)}⊗e_{1}^{T}\right)u,u_{y}=\left(I_{n}⊗e_{1}^{T}A_{y}^{\left(1\right)}\right)u,\\ v=\left(I_{n}⊗e_{1}^{T}\right)v,v_{x}=\left(A_{x}^{\left(1\right)}⊗e_{1}^{T}\right)v,v_{y}=\left(I_{n}⊗e_{1}^{T}A_{y}^{\left(1\right)}\right)v \end{array}$$

For the boundary condition when x=a,y=b, simply substitute $e_{1}$ with $e_{n}$.

By applying conditions to the four boundaries of the board, it can be assembled into

(38) $$\left[\begin{array}{cc} B_{u} & B_{v} \end{array}\right]\left[\begin{array}{c} u\\ v \end{array}\right]=\left[\begin{array}{c} 0\\ 0 \end{array}\right]$$

among them, $B_{u},B_{v}$ are matrices that are respectively assembled from all boundary points.

By applying a matrix transformation to Equation (38), we can derive

(39) $$B_{b}U_{b}+B_{i}U_{i}=0$$

in

(40) $$\begin{array}{c} B_{b}=\left[\begin{array}{cc} B_{u}Z_{b}^{T} & B_{v}Z_{b}^{T} \end{array}\right],B_{i}=\left[\begin{array}{cc} B_{u}Z_{i}^{T} & B_{v}Z_{i}^{T} \end{array}\right] \end{array}$$

The displacement components pertaining to boundary points can be derived utilizing Equation (36)

(41) $$U_{b}=-B_{b}^{-1}B_{i}U_{i}$$

By substituting it into Equation (30), we can derive the result

(42) $$\left(L_{i}-L_{b}B_{b}^{-1}B_{i}\right)U_{i}=\omega^{2}\left(M_{i}-M_{b}B_{b}^{-1}B_{i}\right)U_{i}$$

## 4. Numerical Study

In the numerical exemplification, the in-plane functionally graded material (FGM) rectangular plate comprises a metal–ceramic composite, where the metal component has material parameters: $E_{m}=122.7\;\mathrm{GPa}$, $u_{m}=0.2888$, $\rho_{m}=4420\;\mathrm{kg}/m^{3}$; likewise, the ceramic component also exhibits the same parameters: $E_{c}=132.2\;\mathrm{GPa}$, $u_{c}=0.333$, $\rho_{c}=3657\;\mathrm{kg}/m^{3}$.

However, it is crucial to note that the FGM material properties vary continuously along a predefined gradient according to Equation (1). In employing the differential quadrature method, the nodes are strategically chosen as the second kind of Chebyshev points, ensuring optimal discretization for the analysis. The second class of Chebyshev points are as follows:

(43) $$\xi_{i}=\cos\left(\frac{i}{n}\pi \right),i=0,1,\cdots ,n$$

To facilitate the comparison of numerical results, this article introduces dimensionless parameters and variables

(44) $$\begin{array}{cc} s & =\frac{a}{b},\;\lambda =\frac{\rho_{c}\left(1-\mu^{2}\right)}{E_{c}},\;K_{npq}=\frac{\lambda a}{\rho_{c}}k_{npq},\\ K_{spq} & =\frac{\lambda a}{\rho_{c}}k_{spq},\;\Omega =\omega a\sqrt{\rho_{c}\left(1-\mu_{c}^{2}\right)/E_{c}} \end{array}$$

### 4.1. Results of Vibration Frequency of Unidirectional FGM Rectangular Plates at Different Boundary Confinement and Aspect Ratios

Considering a metal–ceramic in-plane functionally graded rectangular plate, the material property parameters change along the directional functional gradient, and the volume fraction of the ceramic material changes according to the Formula (2).

Table 1 gives the results of the first three-order frequencies of the in-plane vibration of C-C-C-C and C-F-C-F in-plane functionally graded rectangular plates and different aspect ratios. The material parameter properties can be found in the literature [39]. As evident from Table 1, as the number of nodes escalates, there is a notable enhancement in numerical accuracy, ultimately culminating in a convergence result when m=n=15∼21. Consequently, this article employs the specific node count at m=n=17 to delve into the in-plane vibration frequency of FGM rectangular plates.

Table 2, Table 3 and Table 4 present the outcomes related to the first 10-order in-plane vibration frequencies of FGM rectangular plates, configured as S1-C-S1-C, S1-S1-S2-S2, and S2-F-S2-F, under varying elastic constraints. Specifically, for the S1-C-S1-C type FGM rectangular plate, its stiffness coefficient is $K_{nx0}=K_{nx1}=K$, while the other stiffness coefficients are ∞. In the case of the S2-F-S2-F type FGM rectangular plate, its stiffness coefficient is $K_{sx0}=K_{sx1}=K$, $K_{nx0}=K_{nx1}=\infty$, $K_{ny0}=K_{sy0}=K_{ny1}=K_{sy1}=0$. Upon comparing the numerical outcomes with those presented in the literature [39], it is evident that the numerical solution procured through this methodology closely aligns with the solution documented in the literature [39], thereby demonstrating its efficacy in accurately predicting the internal vibration frequencies of unidirectional FGM rectangular plates subjected to diverse elastic constraints.

### 4.2. Results of In-Plane Vibration of Bidirectional FGM Rectangular Plates Under Various Elastic Limits

To further substantiate the efficacy of this approach, we analyzed the impact of varying aspect ratios *s*, gradient parameters *p*, and elastic constraints *K* on the fundamental in-plane vibrational frequency of the bidirectional functionally graded material FGM rectangular plate. Additionally, the volume fraction of ceramic materials changed in a function (3) manner.

**Figure 3:**
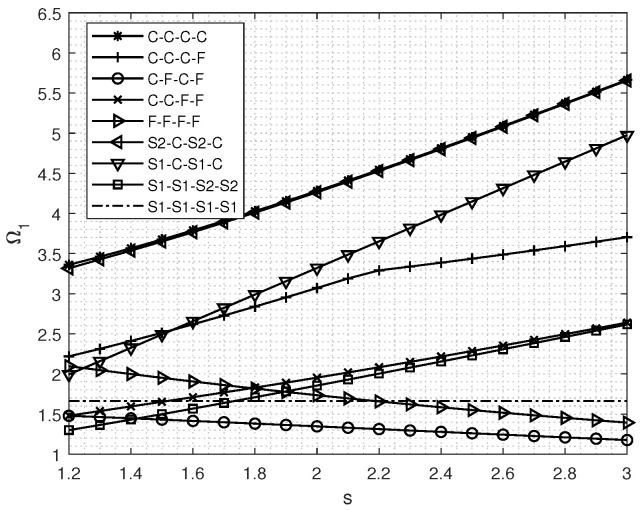
Natural *f* sequence *y* parameter $\Omega_{1}$ of in-plane free vibration for FGM rectangular plates for various aspect ratios.

Figure 3 demonstrates the influence of varying aspect ratio *s* on the fundamental frequency of vibration in FGM rectangular plates; as discernible from the figure, an increase in the aspect ratio *s* leads to a gradual augmentation in the fundamental frequency for S1-C-S1-C and S1-S1-S2-S2 configurations of FGM rectangular plates. Conversely, for C-F-C-F and F-F-F-F types of FGM rectangular plates, there is a progressive decrease in the fundamental frequency. Notably, the fundamental frequency remains constant for S1-S1-S1-S1 type FGM rectangular plates, regardless of the aspect ratio.

**Figure 4:**
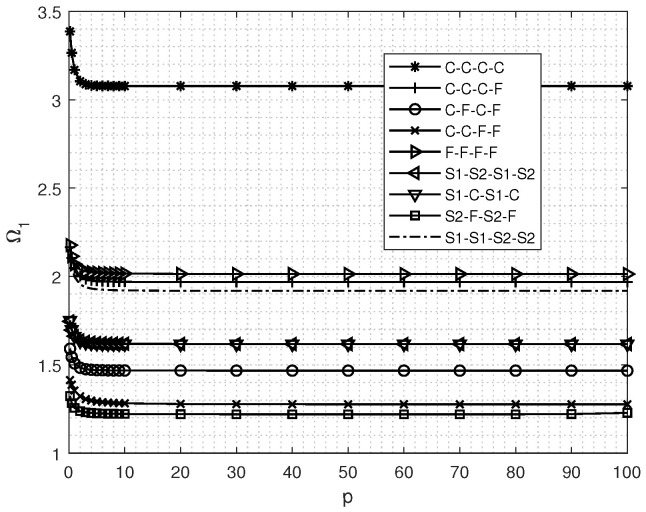
Natural *f* sequence *y* parameter $\Omega_{1}$ of in-plane free vibration for rectangular plates of different *p*.

Figure 4 illustrates the influence of the gradient parameter *p* on the fundamental vibrational frequency within the surface of the FGM square plate. As *p* varies within the range of [0,10], a gradual reduction in the fundamental frequency is observed, attributable to the diminishing overall stiffness of the FGM square plate. When p≥10, the fundamental frequency remains largely stable, signifying that the overall stiffness of the FGM square plate degrades to a level comparable to that of a metal plate.

**Figure 5:**
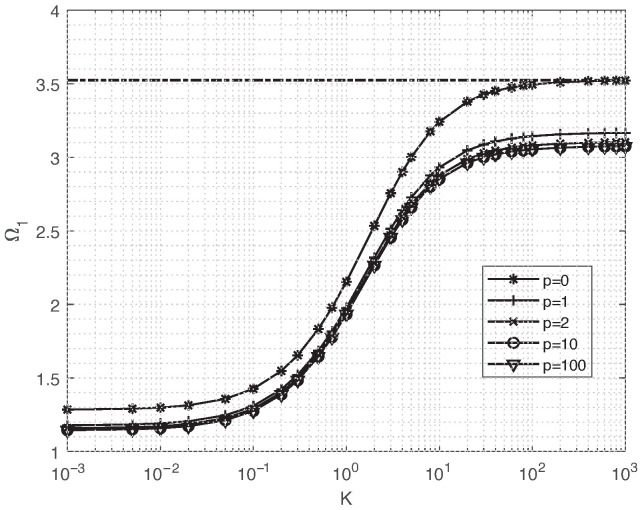
Natural frequency parameter $\Omega_{1}$ of in-plane free vibration for square FGM plates for various elastic restrain boundaries.

Figure 5 illustrates the fundamental frequency variation curves pertaining to FGM square plates, subject to varying elastic boundary constraints *K* and different gradient changes, where the parameter *K* ranges within the interval of $\left[10^{-3},10^{3}\right]$. Upon Figure 5, it becomes evident that as the elastic stiffness *K* escalates, the natural frequency of these plates gradually intensifies. When *K* attains a specific threshold value ($K=10^{3}$), the elastic boundary undergoes a transformation into a rigid boundary, resulting in the convergence of the natural frequency to that of a C-C-C-C plate.

### 4.3. In-Plane Vibration Analysis of Free-Vibration Modal Performance

To reveal the vibration mechanism of in-plane vibration of a rectangular plate, it is of great importance to study the characteristics of in-plane vibration modes.

**Figure 6:**
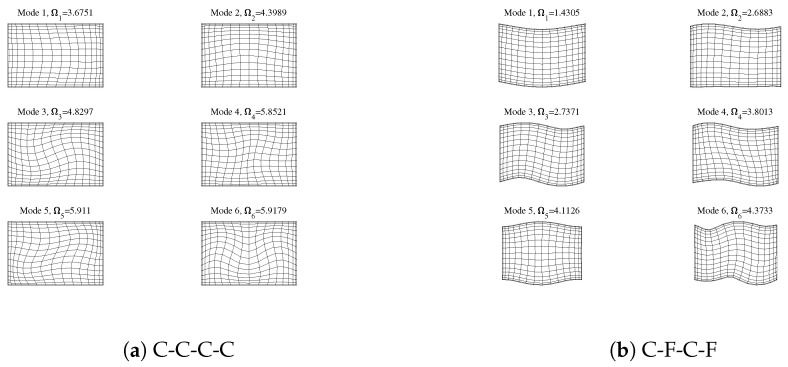

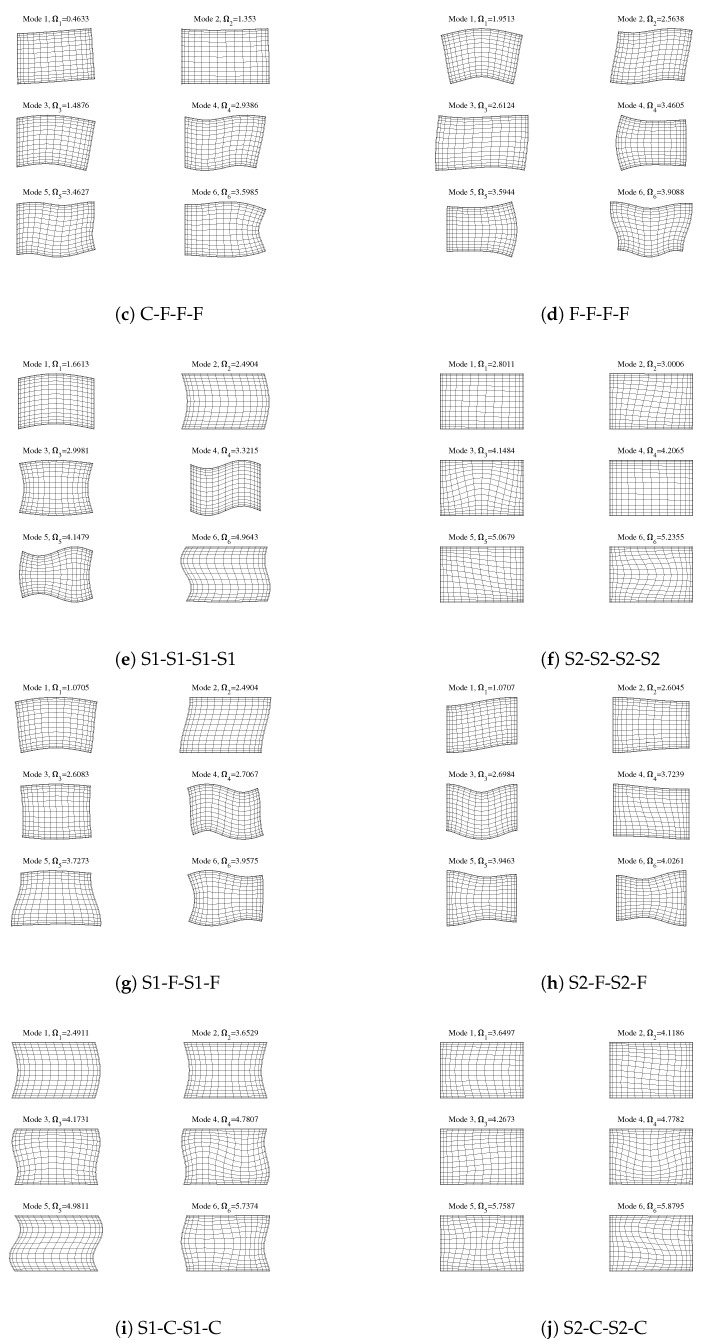
The in-plane free vibration modal shapes of rectangular plates under various boundary conditions are as follows: (**a**) C-C-C-C; (**b**) C-F-C-F; (**c**) C-F-F-F; (**d**) F-F-F-F; (**e**) S1-S1-S1-S1; (**f**) S2-S2-S2-S2; (**g**) S1-F-S1-F; (**h**) S2-F-S2-F; (**i**) S1-C-S1-C; (**j**) S2-C-S2-C.

Figure 6 shows the first six-order vibration mode diagrams and corresponding frequencies of a rectangular plate under various boundary conditions, where the aspect ratio of the rectangular plate is s=1.5. Taking Figure 6a as an example, when all four sides are fixed, it can be seen from the mode diagram that there is neither normal displacement nor tangential displacement on the boundaries, and at the internal nodes, a coupled vibration form exists where normal vibration and tangential vibration occur simultaneously. The rules for other forms of boundary conditions are the same. It can be seen from Figure 6a–j that the in-plane vibration of the rectangular plate is in a state of coupling between normal vibration and tangential vibration.

## 5. Conclusions

Based on the two-dimensional elasticity theory and the variational principle, the differential quadrature method and the Taylor series are used to discretize the differential operator and the integral operator into matrix forms, respectively. This paper is mainly devoted to three aspects: (a) A very effective differential quadrature method for the variational weak form is proposed, and the in-plane vibration frequencies of isotropic rectangular plates are analyzed. (b) The discretization of the dynamic equation is directly derived through the weak form, without involving any transformation and the discretization of high-order derivatives in the strong-form equation. (c) By using matrix transformation, the displacement component parameters related to internal nodes and boundary points are further transformed into an eigenvalue problem of matrices.

The influences of various classical boundary conditions, aspect ratios, and different elastic constraints on the in-plane vibration of rectangular plates are considered. The results show that different boundary conditions, aspect ratios, and elastic constraints have different effects on the in-plane vibration frequencies. As the elastic stiffness coefficient increases, the natural vibration frequency gradually increases, which is due to the increase in the overall stiffness of the rectangular plate. For rectangular plates of C-C-C-C, S1-F-S1-F, and S2-C-S2-C types, the overall trend of the natural vibration frequency increases with the increase in the aspect ratio. However, for S1-S1-S1-S1 type rectangular plates, there is no obvious trend in the first two-order frequencies.

Through the study of the fundamental frequency curves of the in-plane vibration of square plates under different normal stiffness $K_{n}$ and tangential stiffness $K_{s}$, it can be found that when $K_{n},K_{s}\to 0$, the fundamental natural vibration frequency of the rectangular plate degenerates to the result of the F-F-F-F plate; when $K_{n},K_{s}\to 1000$, it gradually degenerates to the result of the C-C-C-C plate. In addition, the normal stiffness has a more significant effect on the fundamental frequency than the tangential stiffness. Finally, through the modal analysis of the in-plane vibration of rectangular plates, the in-plane vibration of rectangular plates exhibits the characteristic of coupling between tangential vibration and normal vibration.

By comparing the analysis of the in-plane vibration frequencies of isotropic rectangular plates using this method with the existing literature, the effectiveness of this method is demonstrated. In addition, the results show that this method can effectively solve the in-plane vibration problem of rectangular plates and has a guiding role in engineering applications.

## Figures and Tables

**Table 1:** Convergence behavior of the lowest three frequency parameters $\Omega_{i}$ of in-plane free vibration for FGM rectangular plates (p=1,K=1).

BCs	$\Omega_{i}$	s = 1					s = 2				
		**11**	**15**	**17**	**19**	**21**	**11**	**15**	**17**	**19**	**21**
CCCC	1	3.2807	3.2807	3.2807	3.2807	3.2807	4.4134	4.4135	4.4135	4.4135	4.4135
	2	3.2834	3.2835	3.2835	3.2835	3.2835	5.8971	5.8972	5.8972	5.8972	5.8972
	3	3.8943	3.8944	3.8944	3.8944	3.8944	6.1845	6.1845	6.1845	6.1845	6.1845
CFCF	1	1.5381	1.5493	1.5515	1.5529	1.5538	1.3789	1.3857	1.3871	1.3880	1.3887
	2	2.7594	2.7592	2.7592	2.7591	2.7591	2.7066	2.7166	2.7186	2.7198	2.7206
	3	2.8714	2.8783	2.8794	2.8801	2.8805	2.7890	2.7899	2.7901	2.7903	2.7904

**Table 2:** The lowest ten frequency parameters $\Omega_{i}$ of in-plane free vibration for S1-C-S1-C FGM rectangular plates for various aspect ratios (p=1,K=1).

s	$\Omega_{i}$	1	2	3	4	5	6	7	8	9	10
0.5	[39]	1.5006	2.0951	2.1034	2.8349	2.8424	3.4721	3.5652	3.6217	3.6497	4.0020
	Text	1.5012	2.0952	2.1042	2.8362	2.8427	3.4728	3.5635	3.6225	3.6251	3.9981
1	[39]	2.1060	3.0947	3.6158	3.6724	4.1170	4.8536	5.2148	5.3687	5.3981	5.5368
	Text	2.1067	3.0949	3.6173	3.6734	4.1145	4.8550	5.2145	5.3695	5.3903	5.5364
1.5	[39]	2.8427	4.1687	4.3685	4.9492	5.2981	6.0793	6.2028	6.3183	7.5133	7.7688
	Text	2.8435	4.1699	4.3686	4.9482	5.2985	6.0793	6.1833	6.3192	7.5106	7.7709
2	[39]	3.6315	4.7486	5.7477	6.0733	6.7040	6.9275	6.9539	7.8078	8.1321	8.8012
	Text	3.6323	4.7499	5.7478	6.0723	6.6835	6.9271	6.9554	7.8093	8.1271	8.7985

**Table 3:** Variation results of the first 10-order frequencies of the S1-S1-S2-S2 type FGM rectangular plate in-plane vibration with the aspect ratio (p=1,K=1).

s	$\Omega_{i}$	1	2	3	4	5	6	7	8	9	10
0.5	[39]	1.6187	1.9707	2.2700	2.6677	2.9090	2.9498	3.3460	3.4689	3.6074	4.1781
	Text	1.6198	1.9713	2.2703	2.6686	2.9079	2.9487	3.3417	3.4439	3.6022	4.1842
1	[39]	2.0317	2.5129	3.1588	3.3022	3.9736	4.6050	4.6593	4.9507	5.0404	5.2527
	Text	2.0329	2.5145	3.1579	3.3004	3.9681	4.6034	4.6593	4.9452	5.0346	5.2495
1.5	[39]	2.4345	3.0816	3.5348	4.4321	4.7748	4.9049	5.5222	6.0549	6.3252	6.7446
	Text	2.4360	3.0833	3.5333	4.4292	4.7736	4.9001	5.5157	6.0484	6.3171	6.7427
2	[39]	2.8432	3.6045	3.9840	5.0180	5.5752	5.6825	6.3336	6.5001	6.9714	8.0359
	Text	2.8451	3.6049	3.9836	5.0174	5.5720	5.6784	6.3250	6.4924	6.9639	8.0296

**Table 4:** The lowest ten frequency parameters $\Omega_{i}$ of in-plane free vibration for S2-F-S2-F FGM rectangular plates for various elastic restrain boundaries (p=1,s=1).

s	$\Omega_{i}$	1	2	3	4	5	6	7	8	9	10
0.5	[39]	1.6187	1.9707	2.2700	2.6677	2.9090	2.9498	3.3460	3.4689	3.6074	4.1781
	Text	1.6198	1.9713	2.2703	2.6686	2.9079	2.9487	3.3417	3.4439	3.6022	4.1842
1	[39]	2.0317	2.5129	3.1588	3.3022	3.9736	4.6050	4.6593	4.9507	5.0404	5.2527
	Text	2.0329	2.5145	3.1579	3.3004	3.9681	4.6034	4.6593	4.9452	5.0346	5.2495
1.5	[39]	2.4345	3.0816	3.5348	4.4321	4.7748	4.9049	5.5222	6.0549	6.3252	6.7446
	Text	2.4360	3.0833	3.5333	4.4292	4.7736	4.9001	5.5157	6.0484	6.3171	6.7427
2	[39]	2.8432	3.6045	3.9840	5.0180	5.5752	5.6825	6.3336	6.5001	6.9714	8.0359
	Text	2.8451	3.6049	3.9836	5.0174	5.5720	5.6784	6.3250	6.4924	6.9639	8.0296
∞	[39]	1.5537	2.7605	2.8813	3.1023	3.4483	3.6212	4.5904	4.6983	4.9557	5.1334
1000	Text	1.5507	2.7588	2.8780	3.1052	3.4445	3.6171	4.5819	4.6971	4.9508	5.1226

## Data Availability

The original contributions presented in this study are included in the article. Further inquiries can be directed to the corresponding author.
